# Concept and contents of a voluntary course for medical students' achievement of a basic qualification in patient safety during the practical year of medical studies

**DOI:** 10.3205/zma001228

**Published:** 2019-03-15

**Authors:** Egbert Opitz, Sylvia Heinis, Andreas Jerrentrup

**Affiliations:** 1 Philipps-University of Marburg, Faculty of Medicine, Marburg, Germany; 2 University Hospital of Giessen and Marburg, Business Director, Campus Marburg, Marburg, Germany; 3 University Hospital of Giessen and Marburg, Center for Emergency Care, Marburg, Germany

**Keywords:** patient safety, undergraduate medical education, clinical clerkship, Germany

## Abstract

**Objective: **Regarding the urgent need of qualification in the field of patient safety, the respective education and training were completed by a voluntary course for 10-15 students in their practical year (PY) provided in cooperation of the private University Hospital of Marburg and the Medical Faculty of the Philipps-University of Marburg. At the same time, this course was intended to develop important knowledge for implementing improvements of the current PY teaching as well as revising the curriculum of Marburg in the medium term.

**Project description:** The PY course on patient safety is offered every six months since 2016 and comprises about 80 lessons.

It is based on the principles of shifting simple knowledge transfer to autonomous preparation by the students themselves, of revising already experienced situations of the professional routine, of working with real data of current patients of the PY students, of fostering teamwork, and of applying very deliberately a large combination of methods with numerous interactive types of teaching.

The topics of those 13 course units include the majority of the most important problem categories of patient safety as reported in the literature such as communication, drug safety, diagnostic errors, and handovers as well as methods for systematic identification and analysis of errors. In the context of a project task, the students evaluate by means of the global trigger tool and 10 patient files of their current wards each if harm has occurred in the treatment of these patients. Afterwards, the students elaborate in teams of 2 a fishbone diagram for one case where an avoidable harm had emerged. In this graph, the deficient process, the factors contributing to its development, the safety measures that are already applied in the department as well as suggested improvements of the students are visualized. In the final lesson of the course, the students explain and describe their diagram to a member of the managing board of the university hospital.

Successful participation is confirmed by an official certificate issued by the Medical Center for Quality in Medicine (Ärztliches Zentrum für Qualität in der Medizin) stating that the course meets the level II requirements (“Basic qualification”) of the training concept on “patient safety” of the Germany medical staff.

**Results: **After meanwhile 5 episodes of this course, the whole curriculum obtained a mean score of “very good” based on the standard questionnaire of the Medical Faculty of the University of Marburg. The students perceive an enormous increase in competence regarding the implementation of specific projects to improve patient safety.

Furthermore, the intensive cooperation with the PY students led to conceiving and establishing further 7 PY courses for the benefit of patient safety and consolidation of entrustable professional activities. In combination with experiences gained elsewhere from courses on patient safety, the collected knowledge could be used for a first draft of teaching and education of patient safety during the entire clinical studies that takes into account the local conditions.

**Conclusion:** In the process of anchoring the topic of patient safety in the Marburg curriculum of medical studies, the introduction of an extensive voluntary course in the second four months of the clinical internship (practical year) turned out to have a very positive effect. Supported by the management board of the hospital and the medical faculty, we consider it useful to permanently provide such an extensive course for a group of students who want to early and intensively deal with the topic of patient safety.

## Zusammenfassung

**Zielsetzung: **Angesichts des dringenden Ausbildungsbedarfs im Fach Patientensicherheit sollte das diesbezügliche Marburger Lehrangebot in Zusammenarbeit zwischen dem privatisierten Universitätsklinikum und dem Studiendekanat des Fachbereichs Medizin durch einen Wahlkurs für jeweils 10-15 Studierende im Praktischen Jahr (PJ) ergänzt werden. Gleichzeitig sollte dieser Kurs dazu dienen, wichtige Erkenntnisse sowohl für rasch umsetzbare Verbesserungen des aktuellen PJ-Lehrangebots als auch mittelfristig für eine Umgestaltung des Marburger Curriculums zu liefern.

**Projektbeschreibung:** Der seit 2016 halbjährlich in Marburg durchgeführte PJ-Kurs Patientensicherheit umfasst ca. 80 Unterrichtsstunden.

Er beruht auf den Prinzipien der Verlagerung einfacher Wissensvermittlung in die selbstständige Vorbereitung durch die Studierenden, der Anknüpfung an bereits erlebte berufliche Situationen, der Arbeit mit den realen Daten der aktuellen Patienten der PJ-Studierenden, der Förderung der Teamarbeit und auf dem bewussten Einsatz eines großen Methodenmixes mit zahlreichen interaktiven Unterrichtsformen.

Zu den Themen der 13 Unterrichtseinheiten des Kurses zählen sowohl die Mehrzahl der laut internationaler Literatur größten Problemfelder der Patientensicherheit wie die Kommunikation, Arzneimitteltherapiesicherheit, diagnostische Fehler und Übergaben als auch Methoden zur systematischen Entdeckung und Analyse von Fehlern. Im Rahmen einer Projektaufgabe untersuchen die Studierenden mit Hilfe des Global Trigger Tools und jeweils zehn Akten von Patienten ihrer aktuellen Stationen, ob bei der Behandlung dieser Patienten Schäden aufgetreten sind. Anschließend erstellen die Studierenden in Zweier-Teams für einen Fall mit einem vermeidbaren Patientenschaden ein Fischgrätendiagramm, auf dem der fehlerhafte Vorgang, die zu seiner Entstehung beitragenden Faktoren, die bereits in der betreffenden Klinik erlebten Sicherheitsmaßnahmen sowie Verbesserungsvorschläge der Studierenden visualisiert werden. In der Kurs-Abschlussveranstaltung erläutern die Studierenden dieses Diagramm gegenüber einem Mitglied der Geschäftsleitung des Universitätsklinikums.

Erfolgreiche Teilnehmende erhalten eine offizielle Bestätigung des Ärztlichen Zentrums für Qualität in der Medizin, dass der Kurs die Anforderungen der Stufe II („Grundqualifikation“) des Fortbildungskonzepts „Patientensicherheit“ der deutschen Ärzteschaft erfüllt.

**Ergebnisse: **Der gesamte Kurs wurde auf der Basis des Standardfragebogens des Marburger Fachbereichs Medizin bei allen fünf bisherigen Durchführungen im Median als „sehr gut“ bewertet. Die Studierenden verspüren einen erheblichen Kompetenzzuwachs bezüglich der Umsetzung konkreter Projekte zur Verbesserung der Patientensicherheit.

Darüber hinaus konnten durch die intensive Zusammenarbeit mit den PJ-Studierenden sieben weitere PJ-Kurse, die gleichermaßen der Patientensicherheit und der Festigung anvertraubarer professioneller Tätigkeiten dienen, konzipiert und umgesetzt werden. In Kombination mit den andernorts in Kursen zur Patientensicherheit gewonnenen Erfahrungen konnten die erzielten Erkenntnisse für einen ersten, die lokalen Rahmenbedingungen berücksichtigenden Entwurf eines Lehrangebots zur Patientensicherheit im gesamten klinischen Studium genutzt werden.

**Schlussfolgerung: **Auf dem Weg zur longitudinalen Verankerung des Themas Patientensicherheit im Marburger Curriculum der Humanmedizin hat sich die Einführung eines umfangreichen Wahlkurses im mittleren Tertial des Praktischen Jahrs bewährt. Bei gegebener Unterstützung seitens der Geschäftsleitung des beteiligten Klinikums und der Leitung des Studiendekanats halten wir ein solch umfangreiches Angebot auch dauerhaft für eine Gruppe von Studierenden für sinnvoll, die sich frühzeitig intensiv mit dem Thema Patientensicherheit auseinandersetzen möchte.

## Introduction

After the publication of the report of the US American Institute of Medicine in 2000 entitled „To err is human – building a safer health system” that extrapolated a span between 44,000 and 98,000 avoidable deaths in US American hospitals per year from two trials [1], [2], [3], the topic of patient safety is perceived by a much broader public than after the publication of several previous articles [4], [5], [6], [7], [8], [9], [10], [11], [12], [13], [14], [15], [16].

Since then, the actual amount of avoidable patient harm has been controversially discussed [17], [18], [19], [20], [21], [22], [23], [24], [25], [26], [27], [28], [29], [30], [31], [32]. However, a current review about the results of all studies that have been conducted based on retrospective file analyses revealed that avoidable harm occurs in at least 5% of all hospital stays ([33], here figure 2). Furthermore, a current systematic review mentions an average of 2-3 critical events per 100 outpatient medical consultations and/or patient files of the included studies. Among those, about 4% were associated with severe health risks which corresponds to 1 severe harm per 1,000 medical consultations [34]. Relevant factors contributing to such harm are errors in medication, avoidable nosocomial infections, communication and teamwork failure as well as diagnostic and handover errors [35], [36], [37], [38], [39], [40], [41], [42], [43], [44], [45].

With regard to the mentioned data, not only the patients’ suffering is discussed but increasingly also the significant subsequent costs of avoidable patient injuries [46]. Based on two analyses in OECD countries conducted on behalf of the German Federal Ministry of Health, these costs amount to about 15% of the hospital expenses for injuries of inpatients and up to 3% of the gross domestic product for injuries of outpatients [47], [48]. 

Based on these data, there is meanwhile a broad consensus regarding the significant need to act and to teach the topic of patient safety that was also addressed repeatedly by the students [49], [50]. As a consequence, the initiatives to teach patient safety increased during the last years, but still they are not found comprehensively in the German speaking countries or elsewhere in Europe [51], [52].

With this background and with the long-term commitment of physicians of Marburg regarding patient safety [53] as well as the basic curriculum that exists in Marburg for about 10 years now, we decided in 2015 to establish an intensive voluntary course on patient safety for the second 4 months of the practical year (PY). The course is subtitled “How can I prevent my patients from avoidable harm?” and accordingly the content focuses on important factors contributing to avoid harm as well as solution approaches [54] compared to the modules II and III of a course that has already been established in the context of medical studies of the University of Zurich, Switzerland. 

## Project description

In order to acquaint medical students early and systematically with the problem of patient safety, several curricula and catalogues of learning targets have been developed worldwide [55], [56], [57], [58], [59], [60], [61], [62], [63]. Hereby, generally longitudinal dealing with the topic of patient safety is recommended over all sections of undergraduate medical education (see also [54], [64]), which is our aim, too. But despite best intentions, significant problems might emerge when introducing seminars on patient safety in the first clinical years of medical studies [[52], p. 13f]. So we decided to first organize a voluntary course in the practical year (PY) based on the following pragmatic reflections:

- Rapid realization instead of waiting for several years until the entire curriculum is fundamentally revised
- Possibility to learn from first courses that have been successfully conducted elsewhere [54]
- Collection of important experience that may be included directly in the revision of the current PY teaching range as well as in the midterm restructuring of our curriculum

After a pilot course, the PY course entitled “Patient safety – how can I prevent my patients from avoidable harm?” has meanwhile been conducted for the fifth time by the faculty of medicine in cooperation with the University Hospital of Giessen and Marburg, Campus Marburg. The following basic principles were relevant:

- Shift of simple knowledge transfer to independent preparation by the students themselves (among others by applying modules of the E-learning program ELPAS of the Department of Medical Psychology and Medical Sociology of the University of Freiburg [65]). In this context, the knowledge acquired in the morning is mostly applied and deepened onsite in the afternoon of the same day.
- Following-up already experienced situations of professional life
- Working with real (pseudonymized) data of current patients of the PY students
- Encouraging the experience exchange with nursing staff
- Fostering teamwork, which is essential in the field of healthcare but only rarely taught (among others promoting interdisciplinary courses, working on team tasks in the E-learning phase, lectures given by professional pilots on principles of increased safety in aviation as well as final presentations that have to be prepared and presented by the teams)
- Intentional implementation of a method mix that is appropriate for the respective lesson, e.g. interactive teaching with the application of audience response systems, simulation patients and role playing in realistic environments [66]
- Organization as voluntary course with 10-15 students each

Overall we attempt to provide the possibilities of independent, active and deep learning with lasting effect based on these principles and combined with the selection of suitable course achievements [[67], p. 12].

Successful participants receive an official certificate issued by the Medical Center for Quality in Medicine (Ärztliches Zentrum für Qualität in der Medizin, ÄZQ) stating that the course meets the level II requirements (“basic qualification”) of the training concept of “patient safety” of the German Medical Association (Bundesärztekammer), the National Association of Statutory Health Insurance Physicians (Kassenärztliche Bundesvereinigung) and the ÄZQ [59]. Since one precondition for this qualification level is professional experience, the course addresses students in the second four months of the PY. At the same time, there is a time distance to final exams so that the students are willing to focus on a course of more than 80 lessons of presence and preparation that are not primarily aiming at knowledge that is relevant to the exam.

The topics of the 13 units of the course include the majority of the most important problems of patient safety described in the international literature such as communication, drug safety, diagnostic failure and insufficient handovers as well as methods for systematic identification and analysis of errors. Detailed information on the course units is summarized in attachment 1 , the learning objectives are found in attachment 2 . The important safety aspect of hygiene [68] is taught in an introduction week that is obligatory for all PY students (see below) so that it is intentionally not included in this voluntary course. 

In the context of the course times mentioned in attachment 1 , the PY students are also expected to work on a team task within the E-learning phase, to attend a morbidity and mortality conference in the department where they are currently working, to elaborate a written essay about a conflict in a work situation they have experienced (as basis for the course day on “communication”), to describe the handover process in their own work environment, to work on a project task as well as to present and discuss the results of their project task in the final lesson of the course.

In the final lesson, the students also have the opportunity to suggest an improvement referring to patient safety, *which should be easy to implement*, and to present it to a member of the hospital’s managing board. Two or three months after the course – i.e. before the final exam – the students receive a feedback if and which one of their suggestions for improvement have been implemented or will be realized.

By means of the so-called global trigger tool (a list of about 50 signal words or situations such as for example sudden decrease of the Hb by ≥25%, pneumonia newly emerged in hospital, or readmission in the emergency unit within 48 hours) [69], [70], [71] and 10 patient files each of their current wards, the students investigate as project task if harm occurred in the context of treatment. In the Department of Pediatric Medicine, we refer to the Global Assessment of Pediatric Patient Safety (GAPPS) [72]. In order to assess the avoidability of harm, we apply the same methods that are described in retrospectively conducted international trials mentioned above (for example [4]). As final course performance, the students elaborate in teams of two a fishbone diagram for an avoidable harm they have identified. The failure, the factors leading to its development, the safety measures that have already been taken in the respective department as well as improvements suggestions of the students are visualized in clearly defined colors. 

This approach consisting of investigating data of current patients is also called “prospective clinical surveillance” [73], [74]. It has to leave out the trigger of “readmission within 30 days”, but in contrast to the otherwise usual assessment of earlier cases applying the global trigger tools, it has the advantage that all people involved in the treatment still know the patients, may be interviewed, and can make precise suggestions to avoid harm in similar future situations.

If no avoidable harm can be identified based on the trigger-positive files, the team elaborates a modified fishbone diagram depicting only the safety measures experienced in the department as well as proposals to further increase patient safety.

Intentionally, the atmosphere of the course is similar to continued education and advanced training programs for medical staff. Generally, it takes place once per week in the afternoon from 1 pm to 4 pm in the study center of the faculty of medicine. In order to be able to read all the literature necessary for preparation or to work on the current patient data, the students are released from their PY activities in the morning of the course days. 

In order to meet the needs of the different learning styles, the literature that has to be read obligatorily is provided in conventional folders as well as electronic files so that the students might prepare the lessons. A selection of further literature that we offer to students who are particularly interested in a specific topic is provided as electronic file only and has to be studied apart from the PY activities. 

Another important addition to the course integrated since spring 2017, is the provision of reports from patients and relatives. To read them, is part of the project task for the respective lesson. These reports in which patients and relatives describe their experience with positive as well as negative aspects of an inpatient stay and associated outpatient episodes in their own words, allow another view on events taking place in a hospital that are usually only seen from the medical perspective. Finally, also emotions stimulated by certain measures and behaviors of the hospital staff are better understood in this way. That is why patient reports on an international level are not only included in the courses [75] but also for example read out regularly at the beginning of monthly meetings of the managing board of hospitals [76].

During the whole course, we focus on ensuring the students that the existing safety level in German healthcare institutions is very high. On the other hand, they should be sensitized for those areas where still a clear potential for improvement is seen despite all previous efforts. We point out that there is nearly never “the one and only” measure to turn a dangerous situation into a safe procedure, but that there is often a complex bundle of measures to achieve sustainable success in limited areas if the measures are consequently implemented [77], [78].

With the method of visualizing factors contributing to incorrect processes that is taught in the course, the students learn to handle a tool they may apply as of the next day in their working area, immediately and without any technical devices in order to start own limited projects of improvement. At the end of the course, they receive this “tool” in form of a rewritable poster together with a set of pens and tape.

## Results

Each lesson of the course is evaluated immediately at its end by means of a standardized questionnaire provided by the faculty of medicine. In the final meeting of the course, it is completed by several questions to evaluate the entire course. The feedback gained in this way is based on a 100% participation of the course attendants; the evaluation results of the five courses that have been conducted until now are summarized in table 1 (Tab. 1).

Overall, the course is rated with “very good” on the average. The students report about significant gains in competence regarding the implementation of specific projects for improvement of the patient safety. They consider the written patients’ and relatives’ reports integrated in the course as highly valuable.

Overall it became obvious that the students’ readiness in the second four months of their PY is very high to deal with the topic of patient safety. At this time, most of them are already able to correlate theoretical information with own experience with patients or the daily routine of an inpatient or outpatient ward, and ORs as well as to implement it in this daily routine – of course depending on the own personality and the circumstances found in the respective department.

The conduction of the course was adapted to certain circumstances due to the following reasons:

- Reduction of the obligatory preparatory material so that it is possible to work on it in the time that is available.
- Except for the course day on communication: limitation of the course length to a maximum of three hours because most participants are not able to concentrate for longer times in the afternoon.
- Extension of the students’ own performance by reports about experienced conflicts in work situations, description of the handover scheme in the respective department, or reports about dealing with experienced incorrect processes in order to elucidate the significance of the course contents for the professional life.
- Invitation of a senior physician from the department of psychiatrics for discussion of those potentially severe medication interactions that are indicated by the computer software that is used in the course for verification of the medication of patients of the departments of surgery or internal medicine suffering from underlying psychiatric diseases, too. Hereby it becomes apparent that there are often situations requiring a very special know-how for correct assessment.

Different than initially expected, in most cases, the treating physicians do not perceive proposals for modifications out of these course units on drug safety as criticism of their competence but as very welcome indication.

The interdisciplinary concept of the course unit on communication is highly appreciated by all participants. However, several modifications were needed in order to adapt the contents and the process of the course to the different experiences and knowledge of the medical students and trainee nurses. Meanwhile all participants have to write a report about a conflict in a work situation they have experienced themselves that is provided in an anonymized form. At the beginning of the course day, the participants use an audience response system to select a priority of the reports they want to discuss. All theoretical aspects are then dealt with only referring to the selected reports.

On one hand, we decided to implement the global trigger tool for pragmatic reasons because it was available in German and because appropriate German literature is available for the objective to obtain a basic qualification regarding patient safety.

On the other hand, its application turned out to be very instructive:

- All PY students find “positive” triggers.
- Nearly all PY students discover harm that emerged in their patients.
- Regarding the harm that has occurred in these patients of the own ward, the students have to think about the possible avoidance as well as proposals for improvement for the benefit of the patients.

Since 2017, the participants receive a feedback about 2-3 months after the course which of their improvement suggestions has already been implemented, which one is currently implemented, and which one will be implemented in the future.

- Five of 14 suggestions from the spring course 2017 have already been implemented.
- From the proposals of the fall course 2017, two of 14 have been realized, one has been in the realization process, and other four have been planned to be implemented.

Examples for successful implementation are the realization of the PY students’ access to the patient data in the emergency unit stored in the hospital information system, an improved regulation regarding hygienic storing of work clothes of the PY students during pauses as well as the availability of important information on interactions of anti-infectives in the online portal of the department of hygiene in order to provide this information for hospital physicians as well as for courses on clinical pharmacology.

Finally, it is important that the intensive cooperation with the students of the course on patient safety led to relevant ideas to improve the range of our PY courses and we implemented them in the context of a PY curriculum. Meanwhile we could introduce for all students starting their practical year in Marburg an *obligatory PY introductory* week with the following five courses (except the third four months starting between Christmas and New Year):

- Instruction on the most important standard medical devices (infusomat, perfusor, surveillance monitor, defibrillator with ECG function, and blood gas analysis device). At the same time, the students have to sign an information sheet on the legal issues.
- Exercises regarding hospital hygiene (based on [68])
- Management of chronic wounds
- Introduction into the hospital information system
- Structured handover (based on ISOBAR standards [79])

We achieved feedback rates of more than 90% assessed immediately after the course by means of questionnaires. The courses were rated as “very good” by the large group starting in the first four months of the PY as well as smaller student groups working for the first time in Marburg in the second or third four months.

## Discussion

Based on data available from numerous investigations about the outpatient as well as inpatient area of the healthcare system, there is a high need for action and education to further improve the patient safety worldwide.

During the last years, education initiatives for patient safety have increased, however, they still do not fully cover all German speaking countries (see an overview for Germany, Austria, and Switzerland published by Rosentreter ([51]; pp 59-64) based on an evaluation of the course catalogues of the medical faculties as well as transcripts of the annual meetings of the society for medical education until 2015). A more advanced distribution of courses on patient safety in England, Scotland, Northern Ireland, and Wales that is neither available nationwide, can be retrieved from the anthology of the General Medical Council and the Medical Schools Council [52]. 

Before realizing our course, we visited an open day of the Salford Royal NHS Foundation Trust that received the certificate of “outstanding” of the Care Quality Commission in 2015 (see http://www.srft.nhs.uk/). We further had a vivid discussion with a member of the core team on planning and implementing the award-winning Zurich course on patient safety integrated in clinical studies that is described more in detail in [54]. Both aspects have supported our strategy to introduce the course on patient safety in the practical year of medical studies in Germany, which is – as far as we know – currently the most ambitious course in this field. After successful attendance, the participants receive an official certificate that they meet the requirements of level II (basic qualification) of the educational concept on “patient safety” of the German Medical Association.

The most obvious disadvantage of the voluntary course is the limited number of participants. However, in our opinion, the selected approach turned out to be very successful due to the reasons described in the following (it is a fact that more than 40% of the students working in the respective second four months in Marburg have registered for the course, which is a very high rate for a course that is not counted for the final exam):

- Starting already with the pilot course, it was possible to cooperate closely with the students because they had encountered numerous aspects of the field of patient safety in their daily work.
- In the context of this intensive cooperation, we received relevant ideas to improve the current PY courses and could implement them in a PY introductory week that is obligatory for all students.
- The proposals of the students encouraged us to ask for hospitation of teaching lessons at and cooperation with other universities when we knew about innovative PY courses (e.g. the Faculties of Medicine of Aachen, Freiburg, Hamburg, and Hannover) [65], [80], [81], [82].

In this way, we could provide two other courses since 2016 that are conducted as tandem and that are very popular with the PY students:

- Clinical Reasoning (with lecturers of the Departments of General Medicine, Internal Medicine, and Surgery)
- Guideline-based pharmaceutic therapy of multimorbid patients (based on the disease groups of the FORTA list [83], with lecturers of the Departments of General Medicine, Clinical Pharmacology, and Psychiatrics)

On the basis of the PY course on patient safety, we could collect valuable teaching and learning experiences in the last years regarding 9 of 13 entrustable professional activities [84]. According to a position paper of the German Association for Medical Education (Gesellschaft für Medizinische Ausbildung), the integration of these experiences in the PY is an optimal transition between students’ medical education and physicians’ training [85]. Their inclusion in the obligatory students’ exams of the German master plan of 2020 is planned [86]. 

Due to the close interrelation of the course contents with the current daily routine, we observe an extraordinarily high learning motivation of the majority of the PY students. 

Based on our experience, we are convinced that a course like ours has punctually an immediate effect on the current situation of the patients, which is highly appreciated (as a result of the students’ work with real patient data of their wards, of developing discussions with the treating team, of feedback of relevant potential drug interactions, and of necessary dose adaptations as well as of specific suggestions for improvement submitted by the course participants presented to a member of the hospital management board at the occasion of the final unit in the sense of a modified “safety walk round” ([87], pp. 49f).

The students receive a feedback about the implemented improvements before their final medical exam. In this way, they already see in this early professional phase how they may be effective which is perceived as an important factor of learning success [88] by many of the course participants.

The experiences made up to now with our courses on patient safety as well as with respective courses in Great Britain [52] made it possible to develop a first draft of longitudinally anchoring the topic of patient safety in the Marburg curriculum of Human Medical Studies. In the upcoming discussion about the revision of the curriculum, this aspect will also be included.

Establishing and further developing the PY course on patient safety as well as the other above-mentioned courses, we could benefit enormously from the ideas of others and from the experiences of other course programs (see above as well as [89], [90]). With this background, we are convinced that it is useful and desirable to establish a cooperation of as many as possible medical faculties of German speaking countries. So we decided to provide a respective exchange of ideas and material to all interested, non-commercially lecturing people via the mentioned contact data. The objective could be for example to establish a “living curriculum” with a collection of evidence-based teaching modules accompanied by train-the-trainer courses, similar to the Canadian Patient Safety Education Program [91].

Factors contributing to the good rating of our course might be the release of the students from work in the morning of the course day so that they can prepare the topics. Another aspect is the close and flexible tutoring as well as a very strong motivation of lecturers who are highly committed to contribute to the course on patient safety.

Since Marburg has a very compact faculty of medicine with a central clinical campus and very short ways to reach all important actors, the organization of the described course is probably easier to realize than elsewhere. In addition, our project meets another of the significant success factors which is mentioned in the following description of the example of developing an interprofessional training center: It seems to be an advantage to involve people with longer periods of employment, experiences with implementing similarly complex projects and respective interface competence ([92], p. 17 (author’s translation)). 

The significance of the results described in this article is limited by the fact that a local standard questionnaire was used for evaluation of the courses and not a validated tool approved by trials. Furthermore, no before/afterwards comparison was available with regard to self-estimation of the course participants regarding their competence of implementing specific projects of patient safety.

More than 40% of the students absolving their entire second 4 months of the PY in Marburg participate in the course, however, in total this is less than 10% of all students in the clinical part of medical studies. For all of them, a less time- and staff-consuming approach has to be found to teach the basic competences in the field of patient safety.

Given the support by the managing board of the university hospital and the Dean of Studies, our experiences encourage us to continue providing an extensive course on patient safety for a group of PY students who want to intensively deal with this topic in this early phase.

Our pragmatic approach allows an easy transfer to other medical faculties by only adapting it to the local circumstances, Furthermore, our concept can be well worked up for the German master plan of 2020 that schedules a division of the PY into units of three months [86]. Hereby, it could be possible to provide the course even more frequently (in the second and third quarterly period).

From the 38 learning objectives mentioned in a German catalogue regarding patient safety for medical studies [63], our course includes 28 and together with the PY introductory week even 32. Many of the topics enable the students to include them into their personal experience in the hospital and to understand their responsibility for safe care provision. Four of the six learning objectives that are not dealt with concern legal questions that are included in other courses.

With regard to the CanMEDS roles, in particular the competences of “collaborator”, “medical expert” and “communicator” are taught, which corresponds to the opinion of internists and surgeons concerning the significance of the CanMEDS model and its implementation in the PY education [93].

## Conclusion

The approach of introducing a voluntary patient safety course in the second four months of the practical year dealing with experiences the students have already made in their PY routine turned out to be very positive. It includes the majority of the most important problems of patient safety in 13 course units.

Based on the support by the managing board of the university hospital and the Dean of Studies, we consider it useful to continue providing an extensive course on patient safety for a group of PY students who want to intensively deal with this topic in this early professional phase.

Taking into account the experiences made in other countries, it will be our future task to develop courses integrated in the whole clinical curriculum. In this context, cooperation with as many as possible medical faculties of German speaking countries would be desirable, e.g. based on the example of the Canadian Patient Safety Education Program.

## Competing interests

The authors declare that they have no competing interests.

## Supplementary Material

Overview of the course units of the practical year course on patient safety in Marburg, Germany

Learning objectives of the practical year course on patient safety in Marburg, Germany

## Figures and Tables

**Table 1:**
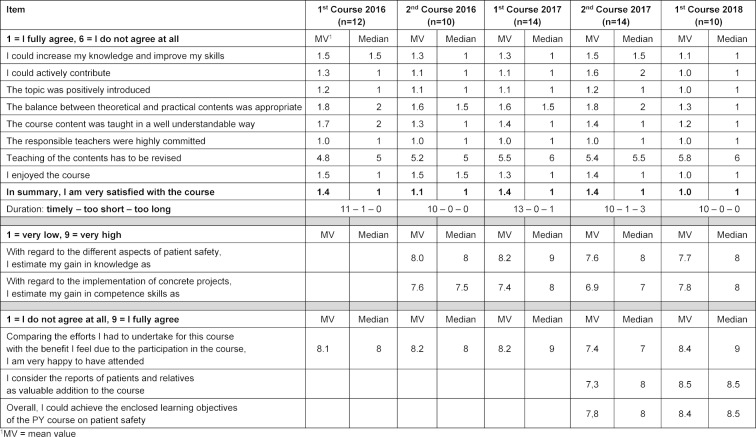
Evaluation results of the Marburg PY course on patient safety
