# Supplementary material for: Concept and contents of a voluntary course for medical students' achievement of a basic qualification in patient safety during the practical year of medical studies
Source: GMS J Med Educ. 2019 Mar 15;36(2):Doc20. doi: 10.3205/zma001228 (PMC6446466; doi:10.3205/zma001228)
Supplement: Overview of the course units of the practical year course on patient safety in Marburg, Germany [file JME-36-2-20-s-001.pdf]

Course units	Duration [h]	Preparation [h]	Contents, description	Particularities
• Introduction and e-learning phase	7.0	1.5	Data on patient safety, failure management, teamwork	ELPAS program (University of Freiburg)
• Drug safety 1+2	4.5	6.0	IT-supported check of the medication of patients with more than 5 active substances regarding potentially severe interactions	**Data of own patients**
• Handover and release management	3.0	3.0	Structured handover in the inpatient area as well as between the inpatient and outpatient sector	ISOBAR scheme,<br>with simulated patients
• Methods of case analysis	1.5	4.0	London protocol<br>Case analysis based on a WHO instructional movie	Use of fishbone diagrams, E-learning (ELPAS)
• Handling severe treatment complications and behavior in case of harm	6.0	4.0	Expectations of patients and relatives after failure-related incidents, consequences for involved physicians, case analysis based on an episode of Grey's Anatomy	Including videos and role plays
• Communication	7.5	2.5	Handling of (own) conflicts, teamwork, speak-up	**With nursing students**
• Diagnostic errors	3.0	3.0	Typical categories of diagnostic errors and strategies to avoid them; situation awareness; training with special databases for diagnosis of rare diseases	With simulated patients; e-learning (ELPAS); database search with terminals of the course participants
• Presentation of a detailed approach of patient safety in an English hospital	1.5	0	Presentation of the strategy to increase patient safety of the Salford Royal NHS Foundation Trust and teamwork on situations regarding patient safety in the own institution	Including current data of the Salford Royal NHS Foundation Trust
• Introduction to the project task and CIRS	3.0	4.0	By means of training files applying the Global Trigger Tool; elaboration of measures based on real CIRS messages	Pediatrics: GAPPS Tool
• Safety training in aviation – lessons to learn for medicine?	3.0	2.0	Safety strategies in aviation presented by an airbus pilot and deduction of lessons to be learnt in medicine	**With nursing students,** with exercises of the pilots' training
**Further performances to render**<br>(partly as basis of the respective course unit)	11.0	1.0	• Working on a team task in the context of the e-learning phase<br>• Visiting a morbidity and mortality conference of the own department<br>• Minutes of a course unit<br>• Written exposé of an experienced conflict in a work situation<br>• Description of the handover scheme in the own working environment<br>• Failure documentation in a teaching ward ("room of horror")<br>• Working on a project task:<br>• Global Trigger Tool-based check of **files of 10 own patients**<br>• Presentation of a failure analysis in the final course event (in teams)<br>• Presentation of a suggestion to improve the patient safety at the University Hospital of Marburg to a member of the managing board	
**Sum [h]**	**51.0**	**ca. 31.0**		

Overview of the course units of the practical year course on patient safety in Marburg, Germany (Status: May 2018)
