# Supplementary material for: Concept and contents of a voluntary course for medical students' achievement of a basic qualification in patient safety during the practical year of medical studies
Source: GMS J Med Educ. 2019 Mar 15;36(2):Doc20. doi: 10.3205/zma001228 (PMC6446466; doi:10.3205/zma001228)
Supplement: Learning objectives of the practical year course on patient safety in Marburg, Germany [file JME-36-2-20-s-002.pdf]

Learning objectives of the practical year course on patient safety

The students prove to have reached the learning objectives marked with * by means of written and oral contributions.

Overall

The student

- has developed a clear position regarding patient safety
- is able to realistically estimate the complexity of approaches and the efforts that are necessary to implement projects for improvement of patient safety
- knows that there is no panacea to solve the problems of patient safety at one go
- knows about successful approaches and projects to improve patient safety with regard to drug safety, communication, diagnostic failure, handover, and release management as well as CIR systems
- has studied patients' and relatives' reports and understood their experiencing of incorrect procedures as well as their wish that the organization learns from those incidents and reacts appropriately with regard to further caregiving
- knows about typical conflicts in the communication with people of a higher hierarchic level as well as the nursing staff and has discussed suitable strategies or promising behaviors
- knows important coping strategies regarding severe treatment complications as well as behavior strategies in case of harm
- knows important safety strategies from aviation as well as an outstanding patient safety project in an English hospital and is able to discuss the transferability of these strategies on the situation in German hospitals
- has participated in a morbidity and mortality conference of the own department and learned about the professional handling of unfavorable procedures
- knows about an established method that enables people to systematically analyze incorrect processes in healthcare management
- knows the approach of prospective clinical surveillance and has applied it in the context of patients' files of the own department
- is able to analyze patients' files by means of the global trigger tool in view of significant warning signals regarding patient safety, to identify and classify patient harm, and to critically rate the results *****
- has identified one incorrect process in the own department by means of the global trigger tool, analyzed it in a team, and visualized the result of this analysis in form of a fishbone diagram including own suggestions for improvement *****
- has presented the analysis of an incorrect process in the own department to experienced physicians of the university hospital and discussed it including own suggestions for improvement *****
- has identified and noted numerous circumstances that jeopardize the patient safety in a small team under time pressure in a "room of horror" based on a simulated patient's performance *****
- has submitted in the final course unit one own specific proposal to increase patient safety to a member of the managing board of the university hospital *****
- experiences him-/herself even in his/her currently low hierarchic position as effective with regard to improving patient safety

Learning objectives of the practical year course on patient safety

- meets all requirements for obtaining the “basic qualification” (level II) of the education concept on “patient safety” of the German Medical Association (Bundesärztekammer), the National Association of Statutory Health Insurance Physicians (Kassenärztliche Bundesvereinigung), and the German Agency for Quality Assurance in Medicine (Ärztliches Zentrum für Qualität in der Medizin) *****

Furthermore, the following learning objectives are pursued in the course units.

Introduction and e-learning phase

The student

- knows the results of the most important international trials on patient safety and is able to estimate the significance of these results for the situation in German hospitals
- knows relevant basic terms to understand the international literature on patient safety and to contribute to projects to improve patient safety
- knows the basic terms and concepts of failure management and successful teamwork
- has analyzed a video clip of a reanimation situation in a small team with regard to incorrect and suboptimal processes and has submitted suitable suggestions for improvement *****

Drug safety

The student

- knows relevant facts of drug safety as well as numerous actors, processes, failure and improvement possibilities that are involved
- knows the FORTA list (Fit fOR The Aged) and knows how it may be applied to optimize medication of own high-risk patients
- has evaluated the medication of multimorbid patients of the own ward containing at least six different substances by means of three types of specialized computer software (for application in general practices and hospitals as well as patients with severely impaired renal function) with regard to potentially severe undesired drug effects and discussed his/her results with two experts (clinical pharmacologist, internist) *****
- knows the problems of frequently false-positive warning signals of software with regard to possible undesired drug effects

Communication

The student

- has contributed to the course contents by writing a report about an experienced situation in which communication problems have occurred *****
- has perceived own typical communication patterns and reflected them
- has dealt with the perception of typical medical communication and behavior patterns by nursing staff, with differences of the physicians’ and nurses’ communication patterns as well as with the strengths of interaction forms of nursing staff and their relevance for patient safety

Learning objectives of the practical year course on patient safety

Diagnostic errors

The student

- knows different types of diagnostic errors and strategies to cope with those failures
- has assigned the events of real cases to different failure types
- has participated in role plays on diagnostic errors in a teaching ward with simulated patients assuming the role of the physician or as observer and has experienced or observed and analyzed typical sources of error *****
- has installed suitable software on the own notebook or smartphone to reduce diagnostic errors, has tested the benefit of the software based on given case reports, is able to apply this software to optimize caregiving to own patients

Handover and release management

The student

- knows different suitable checklists for handover in medical care as well as release management and is able to appropriately apply the ISOBAR scheme in the context of handover *****
- has documented the handover scheme in the own department and if possible submitted a respective suggestion for improvement *
- has participated in role plays of different handover situations in teaching wards with simulated patients in the role of physician or as observer and has experienced or observed and analyzed typical error sources in this context *****
- has participated in a role play on follow-up care by general practitioners after an inpatient stay in a teaching practice with simulated patients in the role of the physician or as observer and has experienced or observed and analyzed typical error sources in this context *****

CIRS (Critical Incident Reporting System)

The student

- knows different medical CIR systems that are applied in Germany including the system used at the own hospital
- has analyzed real reports from a medical CIRS in a small team, has elaborated suggestions for improvement and justified them to a CIRS ombudsman of the university hospital *****

Handling severe treatment complications and stressful courses

The student

- has reported about own experiences or events that have been told with stressful courses as well as the respective coping strategies and/or dealt with examples from the professional life of a very experienced physician
- has acquired knowledge about a specific aid programs for staff members of the own hospital to handle severe treatment complications/stressful courses and the coping strategies

Learning objectives of the practical year course on patient safety

- knows the most important strategies to cope with such situations, is able to interpret the symptoms and to suggest or apply appropriate and suitable strategies for the further procedure, if needed
- has dealt with coping strategies shown in a video regarding severe treatment complications or stressful courses of a physician

Rules of conduct in cases of harm

The student

- has acquired knowledge about the feelings and emotions of patients and their relatives after medical failures and errors as well as the patients' and relatives' wishes about the further procedures after this failure, based on an instruction video with interviews
- has participated in role plays to interact with upset relatives as well as with police officers in a teaching ward in the role of the physician or as observer and experienced or observed and analyzed advisable behaviors *****
- has analyzed in a team incorrect processes shown in an episode of Grey's Anatomy that led to the avoidable death of a patient, has visualized them based on a compiled fishbone diagram and given recommendations to increase the patient safety in the described environment *****

<u>Status</u>: May 12, 2018
